# Influence of Sickle Cell Gene on the Allelic Diversity at the *msp-1* locus of *Plasmodium falciparum* in Adult Patients with Severe Malaria

**DOI:** 10.4084/MJHID.2015.050

**Published:** 2015-08-24

**Authors:** Dilip Kumar Patel, Ranjeet Singh Mashon, Prasanta Purohit, Satyabrata Meher, Snehadhini Dehury, Chhatray Marndi, Kishalaya Das, Bipin Kishore Kullu, Siris Patel, Padmalaya Das

**Affiliations:** 1 Sickle Cell Clinic and Molecular Biology Laboratory, Odisha Sickle Cell Project, Veer Surendra Sai Medical College, Burla, Sambalpur, Odisha, India; 2 Department of Infectious Diseases, Asian Institute of Public Health, Bhubaneswar, Odisha, India

## Abstract

Although several studies have supported that sickle cell trait (HbAS) protects against *falciparum* malaria, the exact mechanism by which sickle gene confers protection is unclear. Further, there is no information on the influence of the sickle gene on the parasitic diversity of *P. falciparum* population in severe symptomatic malaria. This study was undertaken to assess the effect of the sickle gene on the parasite densities and diversities in hospitalized adult patients with severe *falciparum* malaria. The study was carried out in 166 adults hospitalized subjects with severe *falciparum* malaria at Sickle Cell Clinic and Molecular Biology Laboratory, Veer Surendra Sai Institute of Medical Sciences and Research, Burla, Odisha, India. They were divided into three groups on the basis of hemoglobin variants HbAA (n=104), HbAS (n=30) and HbSS (n=32). The *msp-1* loci were genotyped using a PCR-based methodology. The parasite densities were significantly high in HbAA compared to HbAS and HbSS. The multiplicity of infection (MOI) and multi-clonality for *msp-1* were significantly low in HbSS and HbAS compared to HbAA. The prevalence of K1 (*p*<0 .0001) and MAD20 (*p*=0.0003) alleles were significantly high in HbAA. The RO33 allele was detected at a higher frequency in HbSS and HbAS, compared to K1 and MAD20. Sickle gene was found to reduce both the parasite densities and diversity of *P. falciparum* in adults with severe malaria.

## Introduction

Malaria is the major public health problem in India and accounting 1.1 million of reported cases in the year 2011. The number of malaria deaths in subjects aged 5 years or older was higher compared to children younger than 5 years,^1^ that calls for a shifting of malaria control strategies towards adult malaria rather than focusing only on women and children.^2^ There is little doubt that a highly effective vaccine would play a central role in preventing these deaths. This requires a better understanding of the antigenic targets in malaria and the means to overcome the enormous polymorphism of this targets.^3^

The merozoite surface protein 1 (MSP-1) is a leading vaccine candidate antigen and the most abundant surface protein on the blood stage of *P. falciparum*. *msp-1* has extensive genetic diversity^4^. Moreover, it provides multiple effective evasion and drug resistance mechanisms for the parasites and presents a major challenge for the development of an effective malaria vaccine.^5^,^6^ Host polymorphisms like sickle cell gene have been found to influence the population structures of *P. falciparum*, notably in the genes of *P. falciparum* those affect the success and virulence of infection.^7^ In malaria endemic regions, the sickle cell gene has attended high frequency due to its protective effect against severe malaria.^8^–^9^ Both malaria and sickle cell anemia are major public health problems in western part of Odisha. The frequency of sickle cell gene in the study population is 21%.^10^ In a hospital-based study in our institution, we have reported that severe malaria is the second most common cause of death in HbSS subjects.^11^

Surprisingly there is no information on the influence of sickle cell gene on the parasitic diversity of *P. falciparum* population in severe symptomatic malaria in children or adults. Moreover HbSS, a severe form of chronic hemolytic anemia remains a source of great suffering to patients, especially in a developing country like India where the numbers are staggering. Furthermore, when the patients with HbSS get malaria, there will be a superimposed acute hemolytic anemia that become a major cause of death in these patients. Therefore, there is an urgent need to investigate the association, outcomes and mechanism of interaction between HbSS and malaria to provide suitable protection against the potentially fatal threat of *P. falciparum* malaria. Given this we undertook this study to find out the influence of sickle cell gene on the parasitic diversity in the Block 2 region of the *msp-1* in adult subjects with severe malaria.

## Materials and Methods

### Study Area

The study was undertaken at the Sickle Cell Clinic and Molecular Biology Laboratory of Veer Surendra Sai Institute of Medical Sciences and Research, Burla in the state of Odisha, India. This hospital caters the population residing in the western part of Odisha state and the eastern part of Chhattisgarh state. This region has low perennial transmission of malaria with a high frequency of sickle cell gene (21%)^10^ and alpha thalassemia (51%).^12^ The state of Odisha contributes to 23% of positive malaria cases, 50% of *P. falciparum* cases and 15% of malaria-related deaths in India.^13^ In the study area located in western part of Odisha, malaria is the foremost public health problem, and *P. falciparum* accounted for 87.8% of malarial infections.^14^

### Study subjects

Subjects aged 15 years and above, hospitalized in the Department of Medicine, Veer Surendra Sai Institute of Medical Sciences and Research, Burla, between July 2007 to September 2008 and diagnosed to have severe *P. falciparum* malaria, were included in the study. The severity of *P. falciparum* malaria was defined as per WHO criteria.^15^ Severe malaria (SM) was categorized into three sub-phenotypes, (1) Cerebral malaria (CM), (2) Non cerebral severe malaria (NCSM) and (3) Multi-organ dysfunction (MOD).^16^

### Exclusion criteria

Subjects with the following conditions were excluded from the study:

- subjects co-infected with other *Plasmodium* species;
- subjects with other sickle cell syndromes like HbSβ-thalassemia, HbSE, HbSC, HbSD-Punjab;
- children <15 years of age;
- pregnant women;
- subjects who refused to consent.

### Laboratory Investigations

*P. falciparum* examination was made by light microscopy (100 X) of thick blood smears stained by Giemsa. Parasite densities were counted against 200 leukocytes in thick blood films. All the subjects were screened for sickle cell gene by sickling slide test. Those found positive were subjected to agarose gel Hb electrophoresis (pH 8.6) and high performance liquid chromatography (HPLC) using the VARIANT^™^ Hemoglobin Testing System (Bio-Rad Laboratories, Hercules, CA, USA) according to the manufacturer’s guidelines. A complete blood count was done on an automated hematology analyzer (Sysmex pocH-100i; Sysmex Corporation, Kobe, Japan). Biochemical parameters such as serum bilirubin, creatinine, urea, aspartate transaminase (AST) and alanine transaminase (ALT) were done in a semi autoanalyzer (Erba Chem 7; Erba Diagnostics Mannheim GmbH, Mannheim, Germany) as per the manufacturer’s instructions.

DNA was extracted from 5ml of blood by the standard phenol-chloroform method.^17^ Confirmation of *P. falciparum* infection was done by single step polymerase chain reaction (PCR).^18^ Single copy polymorphic gene *msp-1* located on chromosome 9 was analyzed by nested PCR for genotyping^19^. Depending on the variable copy number repeats in block 2 of *msp-1* gene, three distinct allelic families have been described, namely K1, MAD20 and RO33.

The primary PCR was done with 2μL of DNA as a template, using conserved primers for *msp-1*. Family specific primers were used for the secondary reactions with 1μl of the primary PCR product. For *msp-1*, primer pairs specific for each allelic family (K1, MAD20, and RO33) were used. The primer sequences and PCR conditions were as described by Zwetyenga et al.^19^ The PCR-amplified gene fragments of the secondary PCR were electrophoresed on 2% agarose gel and visualized under Gel documentation system (Model: GelDoc XR; Make: BioRad Laboratories, USA) after ethidium bromide staining. This allowed a simultaneous typing of the alleles by size polymorphism and identification of the allelic family.

### MOI and Clonality calculation

The multiplicity of infection (MOI) was calculated by dividing the total number of fragments detected in the individual system by the number of samples positive in the particular system.^20^ Multi-clonality was defined as the percentage of subjects showing more than one genetically distinct parasite type for *msp-1*.

The study was approved by the Institutional Ethical Committee.

### Statistical Analysis

Statistical analysis was done using GraphPad InStat Version 3.00 for Windows. The difference in the MOI in the *msp-1* family that is (K1, MAD20, and RO33) in the three hemoglobin variants (HbAA, HbAS, and HbSS) subjects were made using the Kruskal-Wallis tests. The χ^2^ test was done to compare the prevalence of *msp-1* alleles in the hemoglobin variants and the incidence of various subphenotypes in severe malaria subjects. *P* <0.05 was considered statistically significant.

## Results

**Figure 1:**
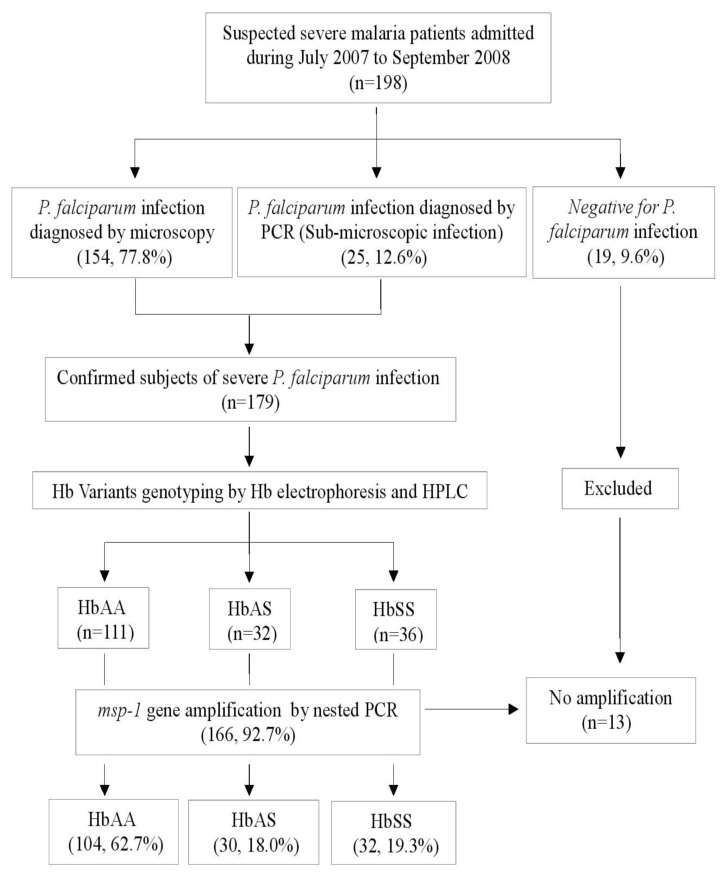
Enrollment and categorization of severe malaria subjects.

Of the 198 blood samples collected with suspected severe *P. falciparum* infection, 166 were finally included in the study (Figure 1). Out of 166 subjects with *msp-1* gene polymorphisms, 55.4% were males. The mean age of the subjects was 31.5 ± 10.3 years. There was no statistical difference in the age, sex, total hemoglobin, total leukocyte count and platelet count in the subjects among the three different hemoglobin variants. The details of the three subphenotypes of severe malaria (CM, MOD, and NCSM) are provided in Table 1. The parasite densities in the three groups HbAA, HbAS, and HbSS were 8966.6±4368.2, 5531.8±3905.7, and 3699.6±2706.1 respectively. Parasite densities were significantly high in HbAA compared to HbAS and HbSS (*p* <0.0001) and did not change with the age of the subjects (Table 1).

All the three reported families of *msp-1* (K1, MAD20, and RO33) were observed among the isolates in the three groups studied. The length variations of *msp-1* amplified products in the three groups have been depicted in Table 2. The multiplicity of infection (MOI) was 3.4, 1.9 and 1.8 in HbAA, HbAS and HbSS respectively. MOI was significantly low (*p*=0.03) in HbAS and HbSS subjects in comparison to HbAA (Table 2).

**Figure 2:**
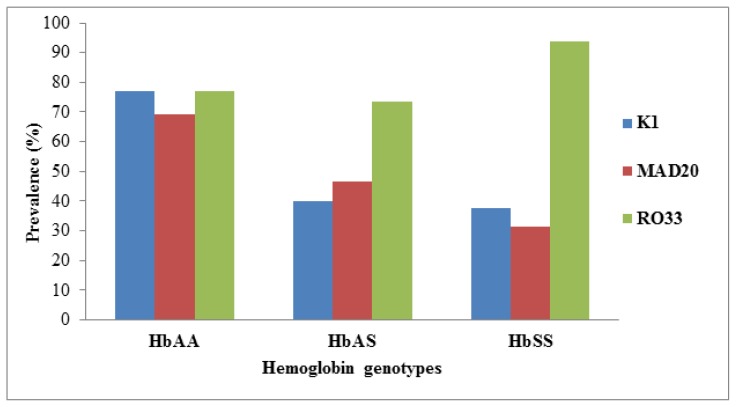
Prevalence of three *msp-1* alleles in three different genotypes (HbAA, HbAS and HbSS). *msp-1*, merozoite surface protein-1; HbAA, normal hemoglobin; HbAS, sickle cell trait; HbSS, sickle cell anemia.

The prevalence of K1 (χ^2^=24.28; *p*<0 .0001) and MAD20 (χ^2^=16.35; *p*=0.0003) alleles were significantly high in HbAA in comparison to HbAS and HbSS subjects, while the prevalence of RO33 was comparable (χ^2^=5.11; *p*=0.0775) in the three groups (Figure 2). K1, MAD20, and RO33 showed more than 2 PCR products as visualized on agarose gel as a double band or multiple bands. The PCR amplification feature of the three allelic families of the *msp-1* gene, as visualized by agarose gel electrophoresis, has been depicted in Table 3. The RO33 was polymorphic and presented in up to 3 size polymorphisms, in some of the HbAA and HbSS subjects.

The proportion of multiclonal isolates in the three groups HbAA, HbAS, and HbSS were 84.6%, 40%, and 37.5% respectively. This multi-clonality was significantly high in HbAA compared to both HbAS (odds ratio [OR], 0.12; *p*<0.0001) and HbSS (OR, 0.109; *p*<0.0001). However, it was similar when compared between HbAS and HbSS (OR, 0.9; *p*=1.0). The combination of clones detected in the *P. falciparum* isolate has been illustrated in Table 4.

None of the *msp-1* polymorphisms (KI, MAD20, and RO33) were over-represented in any of the severe malaria sub-phenotypes (CM, MOD and NCSM) in the three groups of subjects (HbAA, HbAS and HbSS).

## Discussion

We undertook this hospital-based study on 166 subjects in a tertiary care medical center located in western Odisha. The sizable number of adults with severe malaria could be due to the low and markedly seasonal transmission of malaria in this area. In a population with low, erratic and seasonal parasitic transmission most of the people lack acquired antiparasitic immunity (premunition), and symptomatic and severe malaria is found in all age groups including adults^19^. The various subphenotypes of severe malaria that is CM, MOD and NCSM were similar in the three groups of subjects (HbAA, HbAS, and HbSS). In spite of the fact that HbAS confers >90% protection against severe malaria, we encountered a significant number of HbAS subjects with severe *P. falciparum* malaria (30, 18.07%). This could be due to two reasons that are, the prevalence of the sickle gene in the western belt of Odisha state is 21%^10^ and the negative epistasis interaction between the malaria protective effect of alpha thalassemia and HbAS^8^. In a recent cross-sectional study, we found that the prevalence of alpha thalassemia in this region is very high (51%).^12^

The age, sex distribution and baseline laboratory parameters like Hb, TLC and PLT were similar amongst the three groups. The death rate in HbSS was higher in comparison to other two groups, although it did not reach statistical significance. There are several causes of increased mortality in HbSS subjects infected with *P. falciparum* malaria, one of which could be increased the parasitic virulence of individual strains reflected by distinct *msp* alleles.^21^,^22^

The mean parasite densities of microscopically positive samples in the present study were significantly lower in HbAS adults when compared to HbAA. Although several authors have reported low parasite densities in HbAS during episodes of asymptomatic parasitaemia,^23^–^25^ others have reported no influence.^26^,^27^–^30^ In symptomatic malaria, the parasite densities have been found to be low in HbAS compared to HbAA.^26^,^31^ Lower parasite densities during symptomatic infection in HbAS could be due to sequestration of *P. falciparum* in post-capillary microvessels of the brain and other organs, the clearing effect of fever on parasitaemia and the increased level of parasitized HbAS red cells by macrophages.^24^,^26^,^32^

In the four different African studies, undertaken in children with uncomplicated malaria, the parasite densities in HbSS were significantly lower in comparison to that of HbAA and HbAS.^24^,^31^,^33^,^34^ Ours is the only study where we found significant lower parasite densities in adult HbSS subjects with severe malaria when compared to HbAA and HbAS. The various factors that have been found to influence parasite densities are the age of the subjects,^27^ host immune status,^35^ transmission intensity,^19^,^24^ disease phenotypes^30^ and Hb Variants.^19^,^24^,^29^,^34^ In the present study, the variables like age of the subjects, transmission intensity and disease phenotypes were similar in the three groups because all the subjects with severe malaria were of similar age group, and came from the same geographical area with similar transmission pattern. So presumably only the sickle cell gene influenced the parasite densities.

Multiple-strain infections are common in *P. falciparum* malaria that may overwhelm hosts’ immune systems, leading to resource competition amongst the parasite clones that might affect the host morbidity.^36^,^37^ Some studies have found that certain strains of *P. falciparum* population are associated with more virulent infection.^21^,^38^,^39^ Several studies have been undertaken to find out the influence of sickle cell gene on the multiplicity of infection (MOI) in asymptomatic malaria in African children with inconsistent results.^23^,^25^,^28^,^29^,^40^–^42^ In a lone study conducted in Ghanaian children with symptomatic uncomplicated malaria, the authors reported that MOI was lower in HbAS (2.69), and HbSS (2.75) compared to HbAA (3.10). However, this difference was not statistically significant.^34^ The author reported that sickle cell gene had limited influence on the parasite diversity of *P. falciparum.* MOI has been shown to be reduced in clinical malaria due to the anti-parasitic properties of fever and cytokines.^43^ Alternatively lower MOI could be due to reduced parasite densities or simply reflect impaired preexisting premonition or indicate abrogation in symptomatic malaria.^34^ The MOI in the present study was significantly low in HbAS and HbSS genotypes in comparison to HbAA in adult subjects hospitalized with severe malaria. We studied another parameter of parasite diversity that is clonality of *P. falciparum* and found that multiclonal infections were lower in HbAS and HbSS subjects compared to HbAA.

Several authors have reported a positive association between parasite diversity with densities of *P. falciparum* parasite.^23.24,41,44^ The lower MOI and clonality in HbAS and HbSS subjects in our study could be due to low parasite densities due to the inhibitory effect of HbS. Besides this other factors including Hb variants that can influence the parasite diversity are pre-hospital treatment status, age, transmission intensity, genotyping methods, phenotypes of malaria, pregnancy and parity, and immune status. In our study, 90% of the subjects had received pre-hospital treatment with anti-malarial drugs. The mean age was similar, and all the three groups of subjects came from areas with similar transmission pattern. We used the same genotyping method for all the subjects, and none of the subjects was pregnant. All the subjects in the three groups had severe malaria, and the subphenotypes of severe malaria were similar. So the only variable in the three groups which has influenced the parasite diversity is the presence of sickle cell gene in the HbAS and HbSS state.

Studies of *msp-1* allelic family distribution in African children with asymptomatic parasitemia in HbAA and HbAS subjects have reported inconsistent results.^28^,^40^ So far only one study in Gabonese children reported no influence of sickle cell gene on *msp* alleles family. In the present study, the presence of K1 and MAD20 was significantly higher in HbAA in comparison to HbAS and HbSS subjects.^34^ In HbAA, the prevalence of all the three *msp-1* allelic families was similar. However, the RO33 allele was over-represented in HbAS (73.3%) and HbSS (93.8%). The predominance of the certain allelic family in Hb variants could be due to reduced fitness of certain *P. falciparum* strains in erythrocytes containing HbS.^28^

We studied the association of the various allelic families with phenotypes of severe malaria in the three groups of subjects. None of the *msp-1* allelic families was over-represented in any of the severe malaria subphenotypes in the three groups of subjects (HbAA, HbAS, and HbSS). Although some studies have reported over-representation of particular strain/strains of parasites in subphenotypes of severe malaria, these results could not be substantiated further in most of the studies.^21^,^38^,^39^

This study has certain limitations. The use of a single genetic marker (*msp-1*) may underestimate the genetic diversity of infection.^45^ We did not analyze alpha thalassemia in these subjects. Alpha thalassemia influences the susceptibility of *P. falciparum* population.^8^ A prospective longitudinal field study taking into account the various host polymorphisms and including sickle cell gene in a significant number of subjects should be undertaken to overcome these limitations and derive a conclusive result.

In conclusion parasite densities and all the three parameters of parasite diversity namely MOI, clonality and allelic family distribution were significantly reduced in HbAS and HbSS subjects compared to HbAA. Given this provision of early effective anti-malarial treatment during severe illness and chemoprophylaxis of HbSS subjects in malaria endemic area will save valuable lives.

## Figures and Tables

**Table 1:** The Demographic and laboratory data with clinical features of study subjects in the three groups (HbAA, HbAS and HbSS).

		HbAA (n=104)	HbAS (n=30)	HbSS (n=32)	Statistics
Age, years (Mean±SD)		31.00±11.5	32.2±7.2	32.4±8.8	*p*= 0.7201
Sex	Male, (n, %)	57 (54.8%)	16 (53.3%)	19 (59.4%)	
	Female, (n, %)	47 (45.2%)	14 (46.6%)	13 (41.6%)	χ^2^ =0.271; *p*= 0.8732
Parasite densities, /μL		8966.6±4368.2	5531.8±3905.7	3699.6±2706.1	*p* <0.0001
Hemoglobin, g/dL		8.24±2.5	8.9±3.3	7.3±2.4	*p* =0.2187
TLC, 10^3^/μL		8.53±3.43	8.62±2.79	7.8±4.27	*p*=0.7684
PLT, 10^6^/μL		172.2±88.0	175.7±95.9	170.2±70.5	*p*=0.9835
HbS, %		0.0	29.1±12.8	78.2±11.9	*p<0.0001*
HbF, %		0.5±0.7	1.2±0.8	21.4±12.7	*p<0.0001*
CM, (n, %)		34 (32.7%)	5 (16.7%)	10 (31.25%)	χ^2^ =2.931; *p*=0.2309
MOD, (n, %)		31 (29.8%)	10 (33.3%)	14 (43.75%)	χ^2^ =2.148; *p*=0.3417
NCSM, (n, %)		39 (37.5%)	15 (50.0%)	8 (25.0%)	χ^2^ =4.138; *p*=0.1263
Death, (n, %)		11 (10.6%)	2 (6.25%)	6 (18.75%)	χ^2^=2.438; *p*=0.2955
Pre-hospital treatment, (n, %)		96 (92.3%)	26 (86.7%)	27 (84.4%)	χ^2^=2.056; *p*=0.3522

TLC, total leukocyte count; PLT, platelet count; CM, cerebral malaria; MOD, multi-organ dysfunction; NCSM, non cerebral severe malaria

**Table 2:** Distribution of allelic families of *msp-1* in the three groups represented as total number alleles detected, a maximum number of distinct alleles detected and allele fragment size (range in bp).

***msp-1***		**HbAA (n=104)**	**HbAS (n=30)**	**HbSS (n=32)**
	K1	135; 4 (130–600)	18; 2 (115–200)	19; 2 (100–600)
	MAD20	115; 4 (100–500)	18; 3 (170–340)	11; 1 (170)
	RO33	105; 3 (150–400)	22; 1 (150)	29; 3 (150–400)
	**MOI**	**3.4**	**1.9**	**1.8**

*msp-1*, merozoite surface protein-1; MOI, multiplicity of infection; HbAA, normal hemoglobin; HbAS, sickle cell trait; HbSS, sickle cell anemia.

**Table 3:** Polymerase chain reaction amplification products of three alleles (K1, MAD20, and R033) as visualized on agarose gel electrophoresis.

		No amplification (%)	Single length polymorphism (%)	Multiple length polymorphism (%)
**HbAA (n=104)**	K1	23.1	34.6	42.3
	MAD20	30.8	48.1	21.2
	R033	23.1	67.3	9.6
**HbAS (n=30)**	K1	60.0	20.0	20.0
	MAD20	53.3	30.0	16.7
	R033	26.7	73.3	0.0
**HbSS (n=32)**	K1	62.5	18.8	18.8
	MAD20	68.8	31.3	0.0
	R033	6.3	78.1	15.6

HbAA, normal hemoglobin; HbAS, sickle cell trait; HbSS, sickle cell anemia.

**Table 4:** Distribution of clones of the *msp-1* family in *P. falciparum* isolates.

*msp-1* gene polymorphism	HbAA (n=104)	HbAS (n=30)	HbSS (n=32)
K1	8	2	0
MAD20	8	6	2
RO33	0	10	18
K1+ MAD20	8	0	0
MAD20+ RO33	16	2	0
K1+ RO33	24	4	4
K1+ MAD20+ RO33	40	6	8
Multiclonal isolates (%)	84.6	40	37.5
Monoclonal isolates (%)	13.4	60	62.5

HbAA, normal hemoglobin; HbAS, sickle cell trait; HbSS, sickle cell anemia.
